# Clinical evaluation of automated quantitative MRI reports for assessment of hippocampal sclerosis

**DOI:** 10.1007/s00330-020-07075-2

**Published:** 2020-08-04

**Authors:** Olivia Goodkin, Hugh G. Pemberton, Sjoerd B. Vos, Ferran Prados, Ravi K. Das, James Moggridge, Bianca De Blasi, Philippa Bartlett, Elaine Williams, Thomas Campion, Lukas Haider, Kirsten Pearce, Nuria Bargallό, Esther Sanchez, Sotirios Bisdas, Mark White, Sebastien Ourselin, Gavin P. Winston, John S. Duncan, Jorge Cardoso, John S. Thornton, Tarek A. Yousry, Frederik Barkhof

**Affiliations:** 1 Centre for Medical Image Computing (CMIC), University College London, London, UK; 2 Neuroradiological Academic Unit, UCL Queen Square Institute of Neurology, University College London, London, UK; 3 Epilepsy Society MRI Unit, Chalfont St Peter, UK; 4 Universitat Oberta de Catalunya, Barcelona, Spain; 5 Clinical, Educational and Health Psychology, University College London, London, UK; 6 Lysholm Department of Neuroradiology, National Hospital for Neurology and Neurosurgery, UCLH NHS Foundation Trust, London, UK; 7 Department of Medical Physics and Bioengineering, University College London, London, UK; 8 Department of Clinical and Experimental Epilepsy, University College London, London, UK; 9 Wellcome Trust Centre for Neuroimaging, UCL Queen Square Institute of Neurology, University College London, London, UK; 10 Department of Biomedical Imaging and Image Guided Therapy, Medical University of Vienna, Vienna, Austria; 11 NMR Research Unit, Department of Neuroinflammation, UCL Queen Square Institute of Neurology, University College London, London, UK; 12 Radiology Department, Hospital Clínic de Barcelona and Magnetic Resonance Image Core Facility, Institut d’Investigacions Biomèdiques August Pi I Sunyer (IDIBAPS), Barcelona, Spain; 13 Radiology & Nuclear Medicine, VU University Medical Center, Amsterdam, The Netherlands; 14 Digital Services, University College London Hospital, London, UK; 15 School of Biomedical Engineering and Imaging Sciences, King’s College London, London, UK; 16 Department of Medicine, Division of Neurology, Queen’s University, Kingston, Ontario Canada

**Keywords:** Epilepsy, Temporal lobe, Hippocampus, Biomarkers, Magnetic resonance imaging

## Abstract

**Objectives:**

Hippocampal sclerosis (HS) is a common cause of temporal lobe epilepsy. Neuroradiological practice relies on visual assessment, but quantification of HS imaging biomarkers—hippocampal volume loss and T2 elevation—could improve detection. We tested whether quantitative measures, contextualised with normative data, improve rater accuracy and confidence.

**Methods:**

Quantitative reports (QReports) were generated for 43 individuals with epilepsy (mean age ± SD 40.0 ± 14.8 years, 22 men; 15 histologically unilateral HS; 5 bilateral; 23 MR-negative). Normative data was generated from 111 healthy individuals (age 40.0 ± 12.8 years, 52 men). Nine raters with different experience (neuroradiologists, trainees, and image analysts) assessed subjects’ imaging with and without QReports. Raters assigned imaging normal, right, left, or bilateral HS. Confidence was rated on a 5-point scale.

**Results:**

Correct designation (normal/abnormal) was high and showed further trend-level improvement with QReports, from 87.5 to 92.5% (*p* = 0.07, effect size *d* = 0.69). Largest magnitude improvement (84.5 to 93.8%) was for image analysts (*d* = 0.87). For bilateral HS, QReports significantly improved overall accuracy, from 74.4 to 91.1% (*p* = 0.042, *d* = 0.7). Agreement with the correct diagnosis (kappa) tended to increase from 0.74 (‘fair’) to 0.86 (‘excellent’) with the report (*p* = 0.06, *d* = 0.81). Confidence increased when correctly assessing scans with the QReport (*p* < 0.001, *η*^*2*^_*p*_ = 0.945).

**Conclusions:**

QReports of HS imaging biomarkers can improve rater accuracy and confidence, particularly in challenging bilateral cases. Improvements were seen across all raters, with large effect sizes, greatest for image analysts. These findings may have positive implications for clinical radiology services and justify further validation in larger groups.

**Key Points:**

*• Quantification of imaging biomarkers for hippocampal sclerosis—volume loss and raised T2 signal—could improve clinical radiological detection in challenging cases.*

*• Quantitative reports for individual patients, contextualised with normative reference data, improved diagnostic accuracy and confidence in a group of nine raters, in particular for bilateral HS cases.*

*• We present a pre-use clinical validation of an automated imaging assessment tool to assist clinical radiology reporting of hippocampal sclerosis, which improves detection accuracy.*

## Introduction

Hippocampal sclerosis (HS) is the most common cause of temporal lobe epilepsy worldwide [1] and can be effectively treated with surgical excision of the epileptogenic focus [2]. The hallmark pathological features of HS are neuronal loss and gliosis [3], which are characterised on MRI as hippocampal atrophy and T2 signal hyperintensity [4–6]. These qualitative imaging features are used in combination with other clinical data to decide whether surgery is recommended, indicating the central role of imaging in the decision-making process. Importantly, successful seizure-free postoperative outcome depends on precisely identifying and removing the seizure focus [7, 8].

Correct interpretation of MRI findings can be straightforward if the volume loss and increased T2 or FLAIR signal are unilateral and unequivocal. Volume loss assessment can be challenging if the subject’s head is positioned asymmetrically, if the changes are subtle, or if there is some concurrent age-related volume loss. A previous inter-rater agreement study demonstrated a threshold effect at which hippocampal volume difference was only visually detected at a volume asymmetry ratio of 0.7 or lower, meaning many subtle pathological changes could be missed [9]. Assessment of subtle T2/FLAIR signal change can be difficult because the hippocampus, like other components of the limbic lobe (archicortex and periarchicortex), has an intrinsically higher T2/FLAIR signal [10, 11]. When the volume and signal changes are both subtle as well as bilateral, the lack of a clear reference makes a correct diagnosis very difficult if not impossible. Quantification of hippocampal volume and signal intensity [12] as an adjunctive tool to visual assessment has the potential of improving detection accuracy and reducing inter-rater variability.

We have recently proposed a new framework to address key factors for translating quantitative imaging biomarkers from inception to clinical radiology practice [13]. The quantitative neuroradiology initiative (QNI) framework specifies six steps (Table 1). Having identified the appropriate imaging biomarkers (step 1), we developed a dual-algorithm quantification process (step 2). Although hippocampal segmentation in the presence of HS is challenging, recent automated techniques like the Hipposeg algorithm have been sensitive to pathology [14]. These segmentations can then be used for automated quantification of T2 signal in the hippocampus [15]. We developed and technically validated an automated pipeline, combining the two algorithms for the quantification of both hippocampal volume and T2 (qT2) [15, 16]. We encoded the pipeline’s output into a quantitative report (step 3), which includes novel representations of measures or ‘profiles’ along the anterior-posterior longitudinal axis of the hippocampus [17].

**Table 1:** The six steps for imaging biomarker translation outlined by the quantitative neuroradiology initiative (QNI) framework and how each is being addressed in the context of HS

QNI framework step	Application to HS
Step 1—establish the area of clinical need and identify the appropriate proven imaging biomarkers	- Hippocampal volume and qT2 imaging biomarkers for detection of hippocampal sclerosis
Step 2—develop a method for automated analysis of biomarker(s)	- Combination of two algorithms for hippocampal volume and qT2
Step 3—communicate the results via a quantitative report	- Global volume and qT2 values, L:R ratios, and posterior-anterior (P-A) hippocampal graphical profiles contextualised by normative reference data
Step 4—technical and clinical validation of the proposed tool pre-use	- Technical validation has been achieved - Clinical validation in the form of an inter-rater accuracy study is presented in this paper
Step 5—integration of the developed analysis pipeline into the clinical reporting workflow	- Integration into the Picture Archiving and Communication System (PACS) has been achieved. Implementation within a quality management framework is ongoing
Step 6—in-use evaluation	- Future work includes clinical and health economic impact

We are now working towards the introduction of this pipeline into the clinical workflow. This study is a proof-of-concept clinical validation study, representing the clinical pre-use validation (step 4) designed to assess whether the addition of a quantitative report to the neuroradiologist’s workflow enhances detection accuracy and confidence.

We hypothesise that such a quantitative report will (1) decrease inter-rater variability whilst increasing diagnostic accuracy and confidence for determining the presence of HS, and (2) have an identifiable effect across 3 ‘experience levels’ (neuroradiology consultant, neuroradiology specialist registrar, non-clinical image analyst), most pronounced in the less experienced group.

## Methods

### Test dataset

Our study group consisted of 43 subjects who had been scanned on a 3T GE MR750 scanner with a 32-channel coil at our centre. This dataset included patients with HS (15 histologically confirmed unilateral HS; 5 bilateral HS based on consensus of semiology, neurophysiology, and MRI) and 23 age-matched MR-negative epilepsy patients (mean age ± SD 40.0 ± 14.8 years, range 21.1–76.1 years, 22 men).

The imaging protocol consisted of:

1. three-dimensional (3D) T1-weighted inversion recovery fast spoiled gradient recalled echo (3D-T1) sequence for volumetric assessments; field of view (FOV), 224 × 256 × 256 mm (antero-posterior, left-right, inferior-superior); acquisition matrix, 224 × 256 × 256; voxel size, 1 mm isotropic; echo/repetition/inversion time (TE/TR/TI) = 3.1/7.4/400 ms; flip angle 11°; parallel imaging acceleration factor 2;
2. 3D T2-weighted fluid attenuation inversion recovery (T2-FLAIR) sequence; a 3D fast spin echo (FSE) sequence with variable flip angle readout (CUBE); FOV, matrix, and angulation identical to the 3D-T1, but with TE/TR/TI = 137/6200/1882 ms [18];
3. coronal dual-contrast fast recovery fast spin echo proton density/T2-weighted (PD/T2) sequence for T2 quantification; FOV, 220 × 220; matrix, 512 × 512; in-plane resolution, 0.43 × 0.43 mm; 55 slices of 4 mm thickness (TE effective 30 and 119 ms, TR 7600 ms, SENSE factor 2).

### Reference dataset

A normative dataset of 111 healthy controls (age 40.0 ± 12.8, range 17.0–66.6 years; 52 men) was created from subjects on the same scanner and same protocol, as detailed in Vos et al, [17].

### Quantitative report generation and display

Hippocampal segmentation was performed using Hipposeg (http://niftyweb.cs.ucl.ac.uk/) which uses non-linear registration and a template database of 400 epilepsy patients with heterogeneous pathologies [14]. Quantitative T2 maps were generated voxel-wise from the two FSE effective echo time images using a monoexponential fit [15]. A group template was aligned to the long axis of the hippocampus, to calculate cross-sectional volume and qT2 values for slice-wise localisation [16]. The reference data was used to create normative reference ranges for total hippocampal volume, qT2 and left:right total hippocampal volume, and T2 ratios. Additionally, we have created novel hippocampal profiles [17] by producing group templates for the control population, aligning them to the long axis of the hippocampus and calculating cross-sectional area and qT2 for each subject, contextualised with normative reference data.

**Fig. 1:**
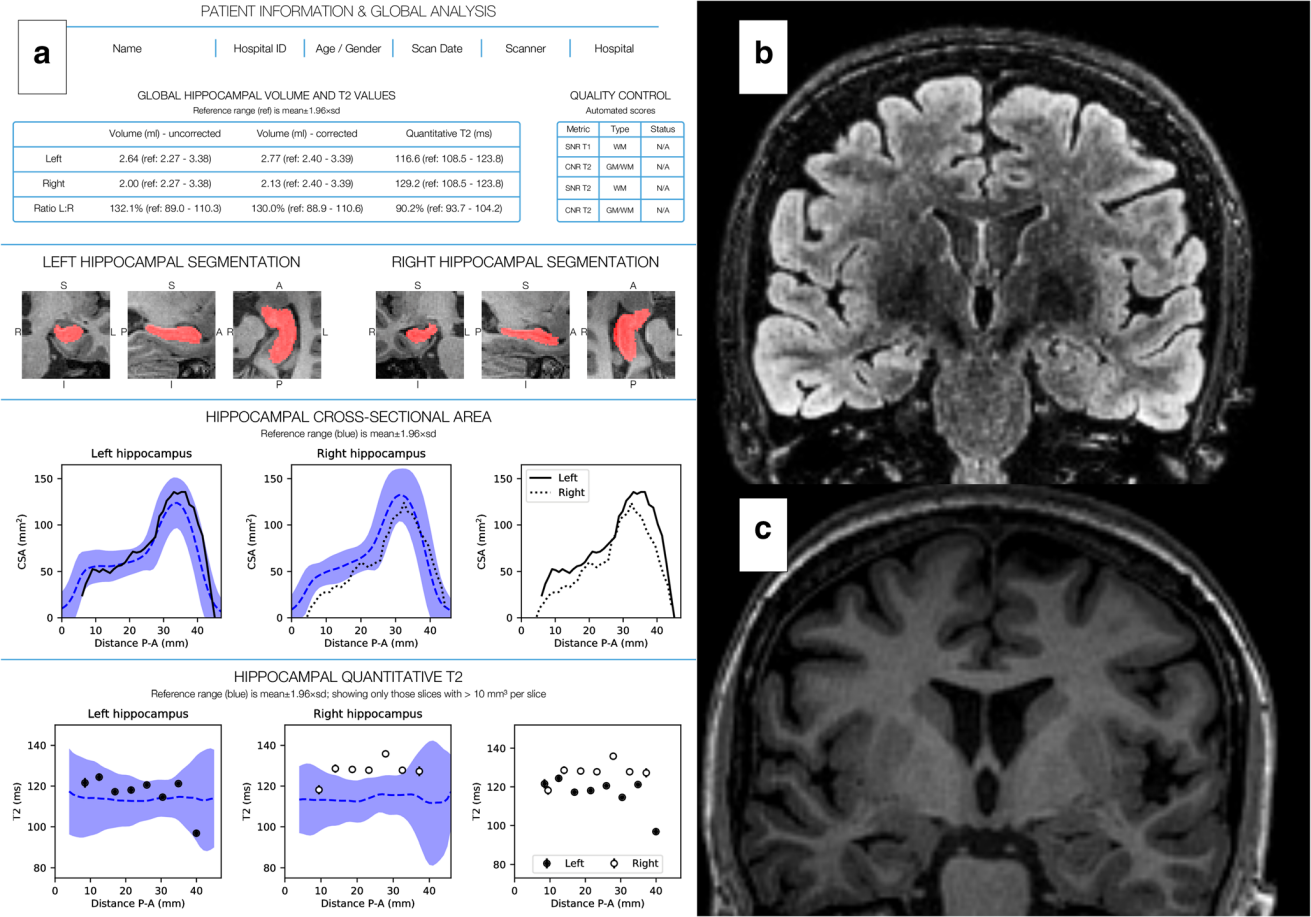
QReport and MR images of a patient with right HS. **a** QReport displaying patient information; global analysis including global measurements and left:right ratios with normative reference ranges in brackets; quality control; snapshots of hippocampal segmentations; graphs for hippocampal cross-sectional area and qT2 posterior-anterior (P-A) along the hippocampal long axis. Graphical display: black lines or dots represent patient’s values, blue dotted line and blue band represent normative data mean ± 1.96SD, graphs with no reference data are a representation of the patient’s left:right ratio. **b** Coronal FLAIR image showing right hippocampal hyperintensity. **c** Coronal T1-weighted image showing right hippocampal volume loss

**Fig. 2:**
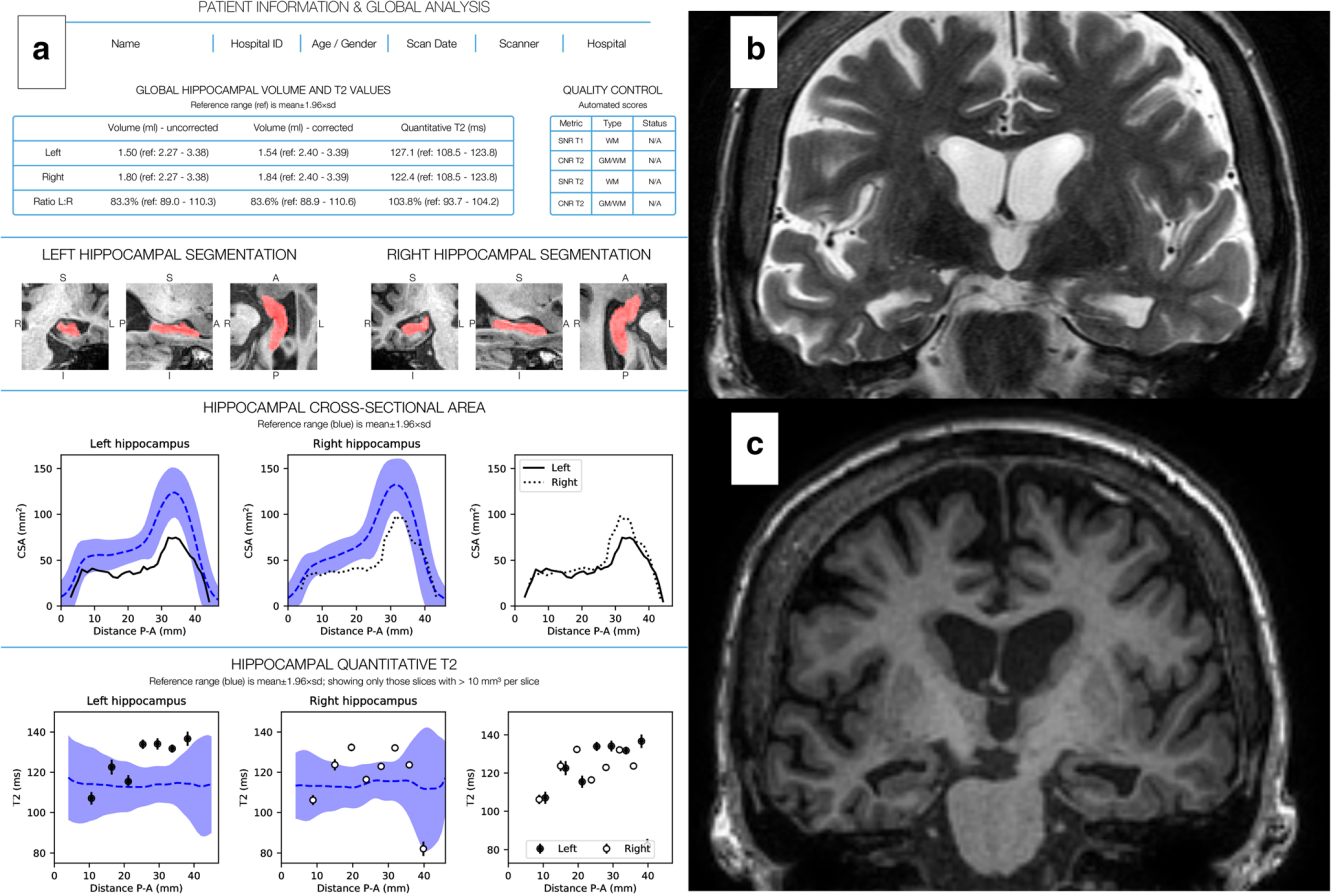
QReport and MR images of a patient with bilateral HS. **a** QReport displaying patient information; global analysis including global measurements and left:right ratios with normative reference ranges in brackets; quality control; snapshots of hippocampal segmentations; graphs for hippocampal cross-sectional area and qT2 posterior-anterior (P-A) along the hippocampal long axis. Graphical display: black lines or dots represent patient’s values, blue dotted line and blue band represent normative data mean ± 1.96SD, graphs with no reference data are a representation of the patient’s left:right ratio. **b** Coronal T2 image. **c** Coronal T1-weighted image

The quantitative report (QReport) displays non-identifying demographics (age, gender, scan date, scanner type, hospital), quality control measures, global volume of each hippocampus as well as hippocampus volume, and qT2 values along its long axis. All values are presented with left:right ratios and normative reference ranges. Snapshots of hippocampal segmentation are displayed (Figs. 1 and 2).

### Assessment task

Three groups of raters were invited to assess the test dataset with and without the QReport available, in a fully randomised order. Each group comprised three raters with a pre-defined level of previous reporting experience: experts (consultant neuroradiologists); trainees (specialty registrars with an interest in neuroradiology); and non-clinical image analysts (MRI radiographers working in neurology centres, non-clinical epilepsy research fellows).

**Fig. 3:**
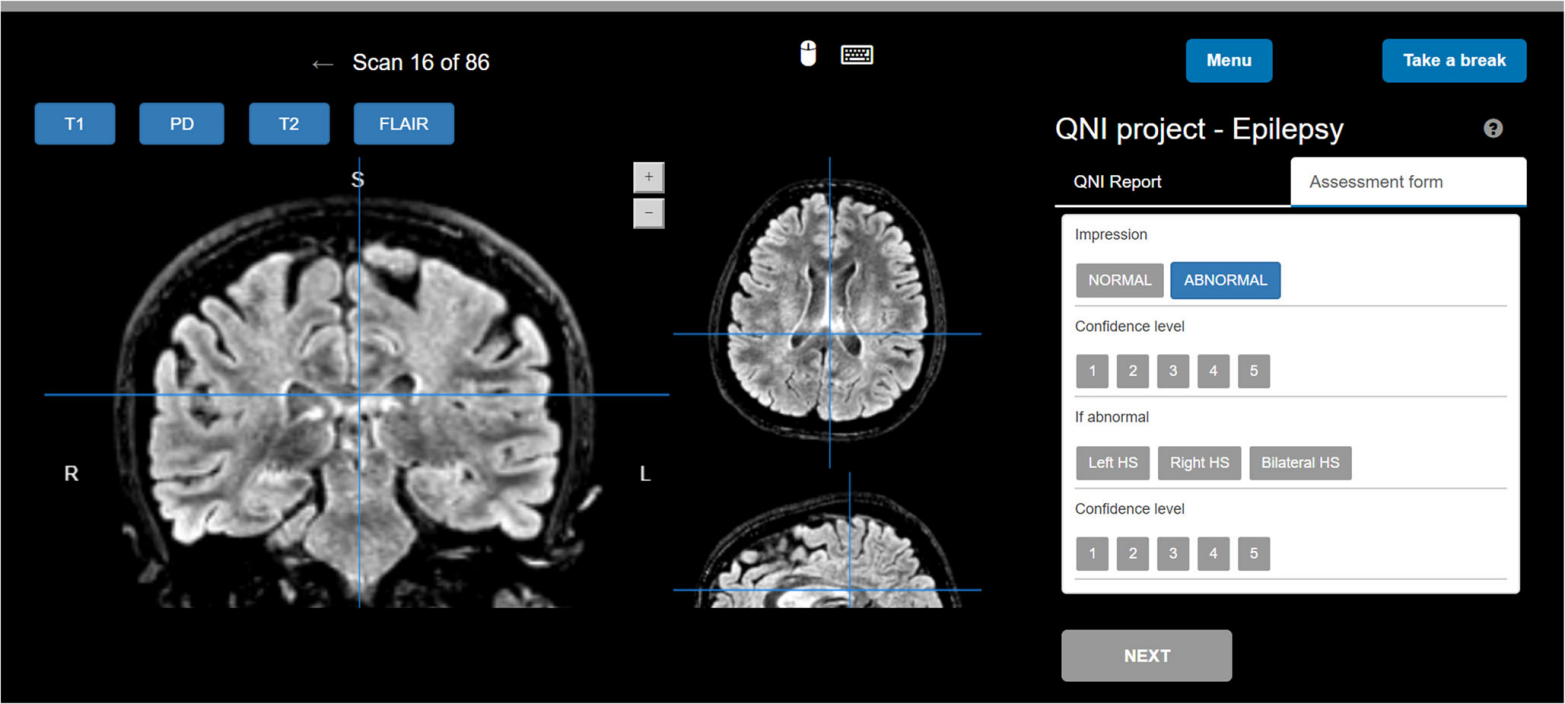
Snapshot of the website platform where raters performed their assessments. T1, PD, T2, and FLAIR sequences were available in interchangeable panels. The assessment form is seen on the right, which was either available by itself or tabbed alongside a QReport

We designed a web platform to facilitate participation from various centres and provide consistent assessment conditions for all raters. The website included instructions for the raters, who were blinded to the diagnosis, followed by the cases displayed in a pre-defined randomly generated order, once with and once without the QReport available (Fig. 3). Each MR study was visualised in three orthogonal planes to mimic the routine neuroradiological environment. Raters were asked to assess each case, stating whether the images were normal or abnormal, and if abnormal, to choose between right, left, or bilateral HS. They were also asked to rate their degree of confidence for both decisions on a scale of 1 (not at all confident) to 5 (extremely confident). The exercise was not timed.

### Statistical analysis

We used signal detection theory tests to determine the effects of the QReport on diagnostic accuracy. Assessments were defined as correctly ‘abnormal’ (true positive, TP), correctly ‘normal’ (true negative, TN), or erroneously ‘abnormal’ (false positive, FP), and erroneously ‘normal’ (false negative, FN). Accuracy was determined as:

$$ \mathrm{Accuracy}=\frac{\mathrm{TP}+\mathrm{TN}}{\mathrm{TP}+\mathrm{TN}+\mathrm{FP}+\mathrm{FN}}\times 100 $$

Data were analysed hierarchically. First, counts were made of correct and incorrect as normal or abnormal against our clinicopathological gold standard, both with and without the QReport, and a McNemar test was applied. Mean accuracy and sensitivity were analysed using paired *t* tests (report present vs. absent). Effect size, Cohen’s *d*, assesses the standardised difference in mean values, and *d* > 0.8 is classified as a large effect size [19]. Cohen’s kappa was used to assess agreement between each rater and the gold standard, a measure which accounts for ‘chance’ agreement [20]. Kappa of 0.60–0.79 can be defined as moderate and 0.80–0.90 as strong agreement [21]. Paired *t* tests were then applied to kappa values (QReport vs. no QReport). The same steps were applied for correct and incorrect lateralisation as R, L, or bilateral HS.

Difference in mean confidence ratings with and without the QReport was assessed with paired *t* tests. In exploratory analyses, mean confidence ratings were calculated for each rater, split by whether the correct or incorrect diagnosis was made and whether the QReport was present or absent. This was analysed using a 2 (correct vs. incorrect) × 2 (QReport present vs. absent) repeated measures ANOVA. We calculated Cronbach’s alpha and intra-class correlation (ICC) as measures of inter-rater agreement and reliability.

All statistical analyses were performed with SPSS Statistics for Mac, Version 24.0. IBM Corp.

## Results

### Test dataset characteristics

The mean age (standard deviation) in years (y) and gender ratio for each group of patients were (a) MR-negative 33.8 y (10.1 y), M:F 13:10; (b) left HS 39.2 (13.5), M:F 3:3; (c) right HS 44.7 y (16 y), M:F 4:5; and (d) bilateral HS 42.3 y (17.3 y), M:F 2:3. ANOVA between HS and MR-negative patients showed no significant age difference (F(1,8) = 1.83, *p* = 0.159). Percentage ratios for volume and qT2 generated by our pipeline for test dataset subjects are presented in Table 2. Values for left and right HS are combined as ‘unilateral’, where volume ratio is calculated as unaffected side:affected side and qT2 as affected side:unaffected side.

**Table 2:** Quantitative characteristics of the test dataset by disease group

Patient group	Volume ratio % (range) normative reference 88.9–110.6	qT2 ratio % (range) normative reference 93.7–104.2
Unilateral HS	72.8 (54.2–89.5)	107.8 (100.3–112.4)
Bilateral HS	86.4 (77.3–98.0)	99.2 (92.6–103.8)
MR negative	97.5 (85.9–110.1)	98.1 (94.9–102.2)

### Detection accuracy

Detection accuracy for all raters was 87.5% without the QReport, yet still showed trend-level improvement with the QReport to 92.5% (*p* = 0.07, *d* = 0.69) (Table 3a). Large magnitude improvement effects were seen in the consultant and image analyst groups (Table 3), and although these did not reach nominal significance, the effect sizes were large [19].

**Table 3:** Correct detection as normal or abnormal, irrespective of lateralisation, by rater group

	Rater group	Without QReport mean (SD)	With QReport mean (SD)	*p* value	Effect size, *d*
Correct designation (normal/abnormal)	Combined	87.3% (4.0)	92.5% (2.2)	0.06	0.73
	1	92.2% (3.6)	96.1% (2.7)	0.30	1.23
	2	85.3% (6.7)	87.6% (3.6)	0.48	0.43
	3	84.5% (15.5)	93.8% (4.8)	0.27	0.81
Sensitivity	Combined	87.5% (13)	90.0% (9.4)	0.25	0.41
	1	96.7% (5.8)	98.3% (2.9)	0.74	0.37
	2	76.1% (16.7)	80% (8.7)	0.50	0.30
	3	90% (5)	91.7% (2.9)	0.42	0.41
Specificity	Combined	87.4% (15)	95.0% (5.7)	0.14	0.54
	1	88.4% (2.5)	94.2% (6.6)	0.18	1.15
	2	94.2% (5)	94.2% (5)	1	0
	3	79.7% (28)	95.7% (7.5)	0.31	0.78
Accuracy	Combined	87.5% (9.0)	92.5% (5.0)	0.07	0.69
	1	92.2% (3.6)	96.1% (2.7)	0.30	1.23
	2	85.9% (6.2)	87.6% (3.5)	0.60	0.33
	3	84.5% (15)	93.8% (4.8)	0.27	0.81

Statistical significance set as *p* ≤ 0.05. *SD*, standard deviation. Rater groups: 1 = neuroradiology consultants; 2 = registrars, 3 = image analysts

Lateralisation accuracy improved with the QReport. When correctly rating a patient’s scan as abnormal, raters made an incorrect lateralisation of the HS (incorrectly choosing right, left, or bilateral) in 8.3% of cases without the QReport and only 3.3% of cases with the QReport. Correct lateralisation of HS by rater tended to increase with the QReport, from 83.5 to 91.5%, *p* = 0.075, with a moderate effect size *d* = 0.68.

For bilateral vs. all unilateral cases, the QReport improved overall accuracy in detecting bilateral cases (*p* = 0.028). Assessment accuracy for bilateral HS significantly increased when using the QReport, mean (SD) from 74.4 (28.77) to 91.1% (17.64), *p* = 0.042, *d* = 0.7.

### Individual rater agreement with the gold standard

Kappa scores increased from 0.74 (SD 0.19), ‘moderate’ to 0.86 (SD 0.09), ‘strong’ with the report across all rater groups for correct lateralisation with a large effect size, *p* = 0.06, *d* = 0.81 (Table 4).

**Table 4:** Kappa scores for agreement of each rater with the gold standard

Rater group	Rater no.	No QReport	With QReport	Net change	*p* value	Effect size, *d*
Experts	1a	0.86	0.82	− 0.04		
	1b	0.93	0.96	0.03		
	1c	0.78	0.96	0.18		
		0.86	0.91	0.05	0.45	0.78
Trainees	2a	0.86	0.82	− 0.04		
	2b	0.69	0.80	0.11		
	2c	0.66	0.74	0.08		
		0.74	0.79	0.05	0.38	0.68
Analysts	3a	0.74	0.93	0.19		
	3b	0.30	0.78	0.48		
	3c	0.93	0.96	0.03		
		0.66	0.89	0.23	0.22	1.13
Total mean (SD)		0.74 (0.19)	0.86 (0.09)	0.12	0.06	0.81

Statistical significance set as *p* ≤ 0.05. *SD*, standard deviation

### Inter-rater agreement

Cronbach’s alpha for agreement across raters showed improvement in overall rating reliability from 0.452 without the report to 0.598 with the QReport, indicating some improved overall reliability. The ICC increased with the QReport from 0.073 to 0.138 for single measures and from 0.417 to 0.591 for average measures, again indicating a small improvement in rater agreement when using the report.

### Rater confidence

Difference in subjective confidence levels reported by raters when assessing scans with and without the QReports was evaluated in a series of paired samples *t* tests (Table 5). These showed that with the QReport, raters were significantly more confident when correctly rating both normal (*p* < 0.01, Hedges’ *g*_z_ = 1.78) and abnormal scans (*p* < 0.01, *g*_z_ = 1.28).

**Table 5:** Rater confidence for normal and abnormal classification for all raters assessed by paired samples *t* tests

Confidence rating	Δ (QReport– no QReport)	SD	95% Confidence interval	t	df	*p* value	Effect size, *g*_z_
Overall confidence	0.35	0.18	0.21–0.48	5.82	8	< 0.01*	1.76
Normal	0.35	0.18	0.21–0.48	5.90	8	< 0.01*	1.78
Abnormal	0.37	0.26	0.17–0.58	4.23	8	< 0.01*	1.28
Correct normal	0.35	0.15	0.24–0.47	6.99	8	< 0.01*	2.12
Correct abnormal	0.32	0.29	0.10–0.54	3.33	8	0.01*	1.00
Incorrect normal	0.14	0.37	− 0.24–0.53	0.96	5	0.38	0.33
Incorrect abnormal	− 0.31	0.24	− 0.69–0.07	− 2.61	3	0.08	− 0.95

‘Correct normal’ refers to the confidence level a rater has indicated when correctly assessing a scan as normal; ‘correct abnormal’ refers to how confident a rater felt when correctly rating an abnormal scan

*Δ* = change in confidence level on 5-point scale. *Denotes statistically significant *p* value ≤ 0.5. *SD*, standard deviation; *df*, degrees of freedom

To assess whether the effects of the QReport on confidence in correct diagnostic decisions depended upon experience level and scan normality, a 2 (QReport/no report) × 2 (normal vs. abnormal diagnosis) × 3 (experience level) mixed ANOVA was run on self-reported diagnostic confidence ratings in *correctly diagnosed* scans. Although power was limited by the small *N*, there was a very large main effect of the QReport, with raters being more confident in their correct diagnoses with the QReport (F(1,6) = 102.65, *p* < 0.001, effect size partial eta squared *η*^*2*^_*p*_ = 0.945). Raters were also significantly more confident in making abnormal diagnoses than normal diagnoses (F(1,6) = 8.911, *p* = 0.024, *η*^*2*^_*p*_ = 0.598), although this was unaffected by the QReport. The QReport’s effects on confidence were moderated by experience level (QReport*Experience Interaction F(2,6) = 7.748, *p* = 0.022, *η*^*2*^_*p*_ = 0.721), indicating a greater confidence increase in the non-clinical image analyst group (F(1,6) = 81.491, *p* < 0.001, *η*^*2*^_*p*_ = 0.931).

## Discussion

We have performed a novel proof-of-concept clinical validation study to determine the effect of the availability of an automatically generated quantitative MRI report for HS on diagnostic accuracy and confidence across 3 levels of experience. Using previously tested algorithms, we developed a novel automated QReport pipeline for hippocampal volume and qT2, and evaluated the benefit of this QReport following a previously proposed scheme [13]. We found that the availability of a QReport increased accuracy and confidence in diagnosing HS, whilst decreasing inter-rater variability, evidenced by strong effect sizes, although not always reaching significance. The thus acquired pilot data will inform a future larger study.

In patients with temporal lobe epilepsy, the correct identification of MR changes typical for HS is central to their management and treatment. This process is often straightforward, but if the changes are subtle, making the correct diagnosis can be challenging. Previous studies using T2 relaxometry, or quantitative T2, have demonstrated high sensitivity and specificity for HS pathology [5, 22, 23], even when there was no obvious loss of hippocampal volume [24]. The importance of the clinical impact as well as the availability of postprocessing solutions led us to the adoption of hippocampus quantification into our QNI framework (Table 1). We have selected techniques that are currently the most suitable for translation into clinical service to support single-subject assessment using clinical quality MRI data. Based on previously published methodology [15, 16], we have encoded a fully automated pipeline, which we combined to create novel graphical representations embedded into a QReport for intended use in the neuroradiologist’s clinical workflow.

Overall, the availability of the QReport led to a large effect increase in assessment accuracy and rater agreement with the gold standard. QReports improved accuracy in all rater groups regardless of prior expertise, and increased correct lateralisation of pathology. Confidence in assessment increased significantly with quantification, consistent with previous outcomes when rating hippocampal atrophy in the case of dementia [25]. Our test dataset represents a broad spectrum of disease severity evidenced by the spread of volume and qT2 ratios (Table 2). Importantly, they included a substantial number of subtle unilateral HS cases with volume ratios > 0.7, a threshold at which unassisted visual detection can be very challenging [9]. We have successfully demonstrated the proof-of-concept for combining single-subject quantification with normative reference data for HS assessment, with potential import to clinical assessment and decision-making.

Previous HS biomarker validation studies have demonstrated enhanced assessment accuracy when using quantitative measures along with visual assessment, or ability to outperform visual inspection. These quantitative measures however have been applied as research paradigms, some using arbitrary thresholds for abnormality [26] and others comparing volume quantification alone to visual assessment alone [27, 28]. Our study presents raters with quantitative information of both volume and T2 signal, allowing them to assimilate the quantitative data with their visual qualitative impressions, as they would do in a clinical reporting setting. This novelty and similarity to the clinical reporting workflow supports a viable translational opportunity for quantitative HS reporting as an adjunct to neuroradiologists’ assessments.

Another important aspect of our study is the use of multiple groups of raters with different experience levels, again reflecting the clinical situation. The largest QReport-associated improvements in both assessment accuracy and confidence were seen in the image analyst group of raters. This aligned with our hypothesis that less experienced raters would benefit from having individual quantified results contextualised within what is expected as normal reference ranges. In addition, we saw large effect sizes for individual rater agreement with the gold standard (kappa) for the expert group of raters. Even more interesting is the finding that the experts’ kappa scores were highest of the three groups without the QReport and they became higher still with the QReport. We assume that raters with higher levels of expertise have built up an internal normative reference based on their own years of practice, which would account for their high baseline scores. The quantitative report would then further assist them in the challenging or subtle cases. Presenting this information to the less experienced raters could level out the baseline discrepancy of expertise and afford the individual patient with a more objective and informed assessment by any imaging specialist.

Interestingly, we saw that the image analyst results improved more than trainees’ with the QReport available. This possibly reflects that image analysts, with no radiological experience, more strongly rely on the report than the trainees, who may struggle to find a balance of integrating the quantitative information with their own assessment in some subtle cases. The improvement in the consultant group indicates that they found a balance between integrating the QReport information where it was helpful.

Our study also addresses the challenging issue of bilateral HS, which can be particularly subtle and difficult to detect visually, making treatment decisions challenging to reach. Despite the small sample size, we found a significant subgroup effect of increased detection accuracy for bilateral HS when a QReport was available. Correct assessment of bilateral HS is clinically very important. Incorrectly diagnosing bilateral HS as unilateral HS, or as normal, could severely impact outcome, as surgical resection of one hippocampus is unlikely to result in seizure freedom postoperatively, whilst likely to cause significant memory impairment. Indeed, it is thought that some surgical failures may be due to a subtle bilateral component that had not been appreciated on imaging [29]. Graphical depiction of subtle raised signal or volume loss along the length of the hippocampus that we provide in our reports may be very useful in helping to elucidate focal abnormalities that are not readily detected visually.

## Limitations

There were several potential limitations to our study. The overall number of subjects enrolled was limited as was the number of raters. Many of the beneficial effects of the QReport were therefore only demonstrated at trend-level significance, albeit with robust effect sizes. Since raters were starting from a high baseline accuracy of detection, a larger test subject population may be needed to demonstrate significant benefit.

Although raters were not informed of the number of positive cases to expect, it is possible that they were primed to expect HS cases at a higher rate than would be encountered in routine clinical practice in which most scans are negative. Contrary to the clinical environment, they were also deprived of any clinical referral data to which they would usually have access.

We also considered the potential for raters to misjudge the QReport. Although we did see instances where a correct assessment was made without a QReport and an incorrect one made with a QReport, this only occurred in 1.7 cases per rater on average, and was even lower for experienced raters at 1.3 cases per rater in the consultant group.

In constructing a dataset with a clinical/pathological gold standard to allow statistical analysis, we may have chosen histologically confirmed or bilateral HS cases with high clinical certainty that were inevitably more visually apparent than more subtle or equivocal cases. This approach is, however, difficult to avoid, if a gold standard is required for reference. Furthermore, our control subjects were MRI-negative patients with epilepsy, and their underlying diagnoses were not established prior to this study. It is possible that subtle hippocampal pathology was present in some of these cases. In addition, although our cohort had a wide age range, it was skewed towards younger individuals, when HS is likely to come to medical attention.

Finally, all data was collected on a single scanner with a uniform imaging protocol. Although providing favourable study conditions, this does not reflect the clinical variability in scanner, imaging protocol, and image quality usually encountered in a radiology department. This variability is a limitation that would need to be assessed and mitigated prior to widespread adoption of our pipeline.

## Conclusion

This proof-of-concept clinical validation represents a key step for the translation of HS imaging biomarkers into clinical practice. We have shown that single-subject quantitative measures, presented in the context of normative data in a novel report format, can improve assessment accuracy, inter-rater agreement, and well-placed rater confidence. Based on the positive results of this study, we now plan to proceed to a supervised introduction into our local clinical service for in-use validation, as well as longer-term outcome and efficiency evaluation to assess the impact on treatment decisions for patients with HS.

## Abbreviations

3D: Three dimensional
ANOVA: Analysis of variance
Corp: Corporation
df: Degrees of freedom
FLAIR: Fluid attenuation inversion recovery
FN: False negative
FOV: Field of view
FP: False positive
FSE: Fast spin echo
GE: General electric
HS: Hippocampal sclerosis
IBM: International Business Machines
ICC: Intra-class correlation
MR: Magnetic resonance
MRI: Magnetic resonance imaging
P-A: Posterior-anterior
PACS: Picture archiving and communication system
PD: Proton density
QNI: Quantitative neuroradiology initiative
QReports: Quantitative reports
qT2: Quantitative T2
SD: Standard deviation
SENSE: Sensitivity encoding
SPSS: Statistical Package for Social Sciences
TE: Echo time
TI: Inversion time
TN: True negative
TP: True positive
TR: Repetition time
y: Years
*η*^*2*^_*p*_: Partial eta squared
